# Are EPA and DHA Derivatives Involved in IBD Remission?

**DOI:** 10.3390/jcm11092388

**Published:** 2022-04-24

**Authors:** Justyna Kikut, Arleta Drozd, Małgorzata Mokrzycka, Urszula Grzybowska-Chlebowczyk, Maciej Ziętek, Małgorzata Szczuko

**Affiliations:** 1 Department of Human Nutrition and Metabolomics, Pomeranian Medical University in Szczecin, 71-460 Szczecin, Poland; justyna.kikut@pum.edu.pl (J.K.); arleta.drozd@pum.edu.pl (A.D.); 2 Department of Pediatrics, Hemato-Oncology and Pediatric Gastroenterology, Independent Public Clinical Hospital No.1, Pomeranian Medical University in Szczecin, 71-252 Szczecin, Poland; mokrzycka.mal@gmail.com; 3 Department of Pediatrics, Faculty of Medical Sciences in Katowice, Medical University of Silesia in Katowice, 40-752 Katowice, Poland; uchlebowczyk@sum.edu.pl; 4 Department of Perinatology, Obstetrics and Gynecology, Pomeranian Medical University in Szczecin, 72-010 Police, Poland; maciej.zietek@pum.edu.pl

**Keywords:** Crohn’s disease, ulcerative colitis, adolescents, resolvin E1, protectin, anti-resolving mediators, inflammatory bowel disease

## Abstract

Recently, an increase in the incidence of inflammatory bowel disease (IBD) has been observed among children and adolescents. Although the pathogenesis of IBD is not fully elucidated currently, actual research focuses on the occurrence of imbalance between pro- and anti-inflammatory molecules and future identification of the role of cytokines in IBD therapy. The purpose of this study was to compare the concentrations of eicosapentaenoic and docosahexaenoic acid derivatives during both phases of Crohn’s disease (CD) and ulcerative colitis (UC). The study included 64 adolescent patients with CD (*n* = 34) and UC (*n* = 30) aged 13.76 ± 2.69 and 14.15 ± 3.31, respectively. Biochemical analysis was performed on a liquid chromatography apparatus. A statistically significant lower concentration of resolvin E1 (RvE1) was observed in the CD group relative to UC. In the active phase of CD, a statistically significantly higher concentration of protectin DX (PDX) was observed relative to remission CD. Comparing the active phase of both diseases, a statistically significantly higher concentration of resolvin E1 (RvE1) was observed in UC relative to CD. Comparing the remission phase of both diseases showed statistically significantly higher PDX levels in CD relative to UC. Our study adds to the knowledge on the involvement of anti-inflammatory lipid mediators in both IBD entities. In conclusion, it seems that the marker differentiating both disease entities in the active phase may be RvE1, while in the remission phase, PDX. In CD remission, the greatest involvement was observed towards PDX, whereas in UC, MaR1, RvE1 and 18RS-HEPE seem to be the most involved in remission.

## 1. Introduction

There is currently a significant increase in the incidence of inflammatory bowel disease (IBD) among children and adolescents. Although the incidence seems to be stabilizing in western countries, still 25% of patients are under 18 years of age [1]. The development of the disease is influenced by many factors including genetic, environmental and diet. An overview of the factors and etiology is provided in our other article [2]. IBD is divided into two main entities, i.e., Crohn’s disease (CD) and ulcerative colitis (UC). IBD is characterized by chronic inflammation associated with damage to the mucosal layer of the gastrointestinal tract [3]. Currently, drug treatment, which is effective in most cases, is associated with impairment of physiological functions of the body, thus causing side effects. Moreover, unfortunately some patients do not respond positively to the proposed treatment, or they lose effective response to treatment over time. Therefore, it is still necessary to search for new possibilities of therapy in the treatment of IBD [4].

**Figure 1:**
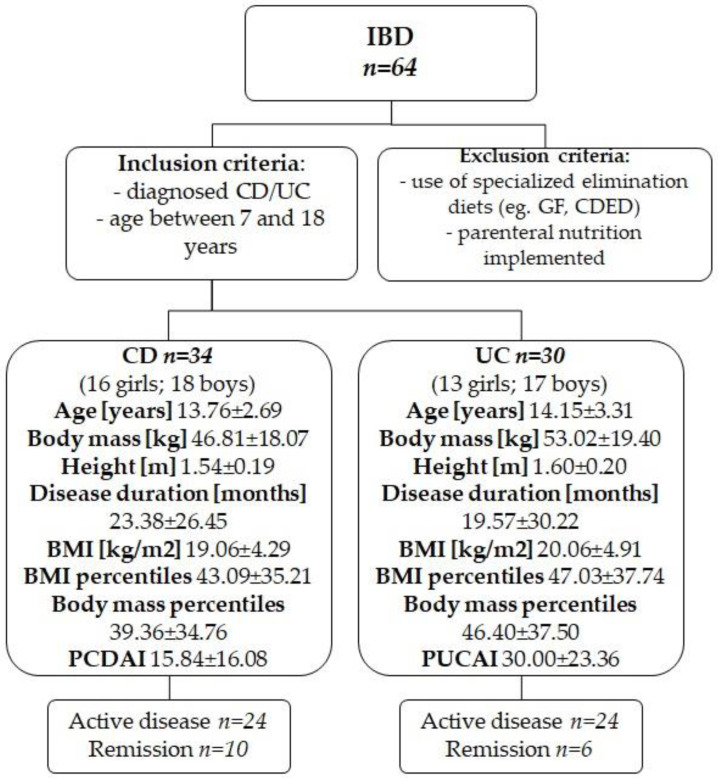
Characteristics of the study group (CD—Crohn’s disease, UC—ulcerative colitis). PCDAI—Pediatric Crohn’s Disease Activity Index; PUCAI—Pediatric Ulcerative Colitis Activity Index; GF—gluten-free diet, CDED—Crohn’s Disease Exclusion Diet; BMI—body mass index; IBD—inflammatory bowel disease. There were no statistical differences between the groups.

Arachidonic acid is a source of omega 6 and omega 3 fatty acids. Both pro- and anti-inflammatory lipid mediators are produced from the omega 6 fatty acid pathway. Polyunsaturated fatty acids (PUFAs) are precursors of eicosanoids, i.e., lipid mediators that are signaling molecules [5]. On the omega 3 acid pathway, anti-inflammatory derivatives of eicosapentaenoic (EPA) and docosahexaenoic (DHA) acids are formed [6]. EPA further produces E-series resolvins (RvE), while DHA produces D-series resolvins (RvD), D-protectins (PD) and maresins (MaR) [7]. Macrophages via DHA produce pro-resolving molecules such as maresins, and maresin 1 is among the first identified from this group [8]. D-series resolvins are also produced in the pathway through the 17-HDHA marker. In turn, 18-HEPE is a marker of the pathway that forms E-series resolvins [9]. The schematic is shown below (Figure 1). Resolvins, protectins and maresins are a group called specialized anti-resolving mediators. The anti-resolving mediators are responsible not only for stop signaling for pro-inflammatory mediators and neutrophil influx but also for removing pro-inflammatory cells and restoring body homeostasis, reducing the severity and duration of inflammation [10]. Thus, the synthesis of proresolving lipid mediators is a response to the activation of inflammation in the body. By modulating the levels of pro-inflammatory cytokines and chemokines and other cells involved in inflammation such as leukocytes and monocytes, the anti-resolving mediators regulate the course and consequently the quiescence of inflammation [11]. The number of studies on the effect of resolvins in humans with IBD is negligible. However, abnormalities in the action and secretion of proresolving mediators impair the silencing of inflammation, consequently leading to the persistence of chronic inflammation in the body [12]. The imbalance between pro- and anti-inflammatory cytokines leads to tissue degradation [13]. Maresins may appear in later stages of inflammation, often during the period of resolution of inflammation [12]. Interestingly, both resolvins and lipoxins are involved in modifying the secretion function of the prototypical T-helper 1 cytokine interleukin 12 (IL-12), which is also a target for biological drugs used for IBD therapy [14].

The aim of this study was to compare the concentrations of anti-inflammatory mediators in IBD to identify the course of inflammation quenching. Consideration of both disease entities (CD, UC) and their phases (active and remission) will distinguish the course of diseases and will allow the introduction of new therapies leading to remission.

## 2. Materials and Methods

Approval from the Bioethics Committee of the Pomeranian Medical University in Szczecin (KB-0012/131/18, dated 26 November 2018) was obtained prior to conduct of the study. Informed consent signed by a parent or guardian was obtained from all study participants. Both legal guardians and minors over 16 years of age provided written consent to participate in the study. Participants were informed about the possibility of opting out of the study.

### 2.1. Characteristics and Division of the Study Group

The characteristics and distribution of the study group are shown in Figure 1. The diagnosis of IBD was confirmed on the basis of endoscopic examination (Olympus, Tokyo, Japan) with histopathological evaluation (material collected during endoscopic examination). Subsequently, an ultrasonographic examination was performed. For patients diagnosed with CD, enteroclysis or enterography with computed tomography (CT) imaging was performed. During the course of the study, 6 participants dropped out for various reasons, representing approximately 10% of the study population. The Pediatric Crohn’s Disease Activity Index (PCDAI) was used to determine disease activity in CD and the Pediatric Ulcerative Colitis Activity Index (PUCAI) in UC.

**Figure 2:**
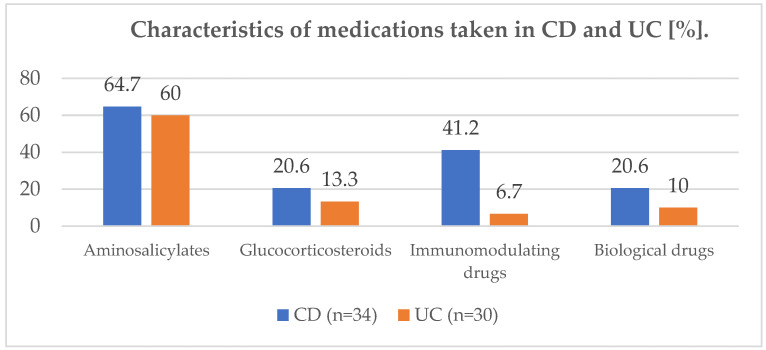
Characteristics of medications taken in CD and UC (%). CD—Crohn’s disease, UC—ulcerative colitis.

**Figure 3:**
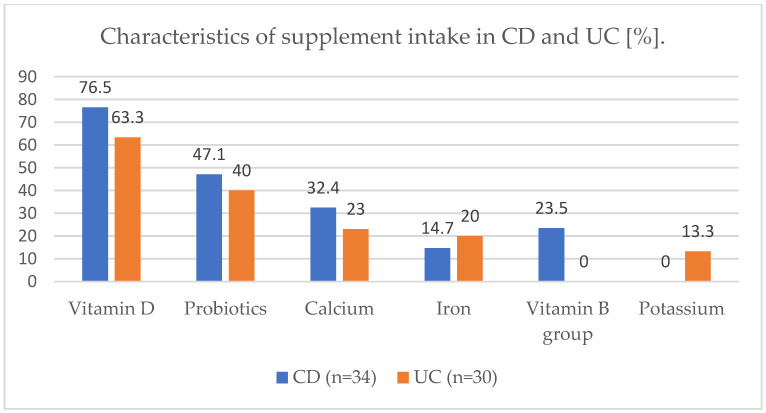
Characteristics of supplement intake in CD and UC (%). CD—Crohn’s disease, UC—ulcerative colitis.

The characteristics of the medication groups and supplementation taken are shown in the figures below (Figure 2 and Figure 3).

### 2.2. Anthropometric Measurements

Nutritional status of the patients was analyzed using anthropometric parameters such as body weight (±0.1 kg) and height (±0.5 cm). A digital physician scale with height rod (Radwag WPT 60/150 OW, Poland) was used for these measurements. The obtained results were compared with OLA and OLAF centile grids (for body weight and BMI), recommended by the “Child Health Center” Institute (Warsaw, Poland) [15].

### 2.3. Sample Collection

Seven milliliters of venous blood were obtained from patients and collected into adequate tubes containing EDTA as an anticoagulant. The blood was then centrifuged and separated into individual Eppendorf tubes for differentiation of morphotic components. The samples were stored at −80 °C in a freezer. The eicosanoid extraction methodology was performed on a liquid chromatography (HPLC) apparatus (Agilent Technologies, Cheshire, UK).

### 2.4. Extraction of Eicosanoids

EPA and DHA derivatives were extracted from plasma using an RP-18 SPE column (Agilent Technologies, UK). EPA and DHA standards used (Cayman Chemicals, Ann Arbor, MI, USA): 18-HEPE (cat no. 3284), 17-HDHA (cat no. 3365), 10(S)17(R)DiDHA (Protectin DX) (cat no. 10008128), Maresin 1 (cat no. 10878), Resolvin D1 (cat no. 10012554) and Resolvin E1 (cat no. 10007848). Starting with the HPLC analysis, 400 µL of serum, 50 µL of PGB internal standard and 1 mL of pre-cooled acetonitrile were added to an Eppendorf tube. Samples were vortexed for 1 min and sequentially incubated at −20 °C for 20 min. The samples were then centrifuged for 15 min (14,000 rpm). In the next step, the supernatant was poured into 7 mL samples, and 4.5 mL of 1 mM HCL was added. To achieve pH = 3, 1 M HCL was added. The next step of the analysis carried out was the conditioning of the column using 100% acetonitrile and 20% acetonitrile (1 mM HCL). The samples were then eluted into Eppendorf tubes with a mixture of methanol and ethyl acetate (1:1). The resulting samples were analyzed on a liquid chromatography apparatus (Agilent Technologies 1260). The dedicated Agilent ChemStation software (Agilent Technologies, Cheshire, UK) was used to control and subsequently analyze the results.

### 2.5. Statistical Analysis

Statistical analysis was performed using Statistica 13.3 software (Statsoft, Krakow, Poland). Normality of distribution was checked using the Shapiro–Wilk test. All data presented a normal distribution. T-student test was used to compare diseases in the active phase. Comparison of active and remission phases and between remission phases of both diseases was performed using the non-parametric Mann–Whitney U test. A value of *p* < 0.05 was taken as the level of statistical significance.

## 3. Results

**Figure 4:**
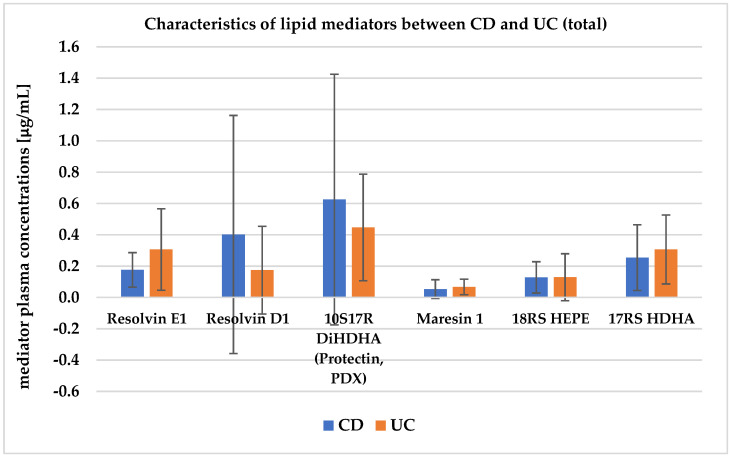
Characteristics of lipid mediators between CD and UC (total). CD—Crohn’s disease, UC—ulcerative colitis.

Statistically significantly lower levels of resolvin E1 were observed in the CD group relative to UC (*p* = 0.014). Additionally, the UC group showed lower concentrations of RvD1 and 10S17R DiHDHA relative to CD. There was no statistical significance between the above mediators due to high standard deviation (SD) values. In contrast, higher levels of Mar1 and 17RS HDHA were observed in the UC group relative to CD. The level of 18RS HEPE was not significantly different between the disease entities (Table 1 and Figure 4).

In the next step, mean concentrations of lipid mediators were compared by active phase of disease and remission. In the CD group, statistically significantly higher PDX levels (*p* = 0.046) were observed in the active phase of the disease relative to remission. In addition, higher levels of RvD1 were observed in the active phase of CD disease. Higher concentrations of RvE1 were demonstrated in CD remission relative to active disease. Levels of 17RS-HDHA, MaR1, and 18RS-HEPE showed no major differences between disease phases (Table 2).

When comparing the active phase of UC with remission, higher levels of RvE1 and 18RS-HEPE were observed in the active phase of UC relative to remission. RvD1, PDX, MaR1, and 17RS-HDHA remained unchanged between UC phases (Table 2).

In contrast, comparing the active phase of the two diseases showed statistically significantly higher RvE1 levels (*p* = 0.011) in UC relative to CD. A higher concentration of 17RS-HDHA was observed in UC in comparison to CD. In contrast, the CD group showed significantly higher concentrations of RvD1 and PDX relative to UC. MaR1 and 18RS-HEPE were slightly lower in the CD group relative to UC (Table 2).

**Figure 5:**
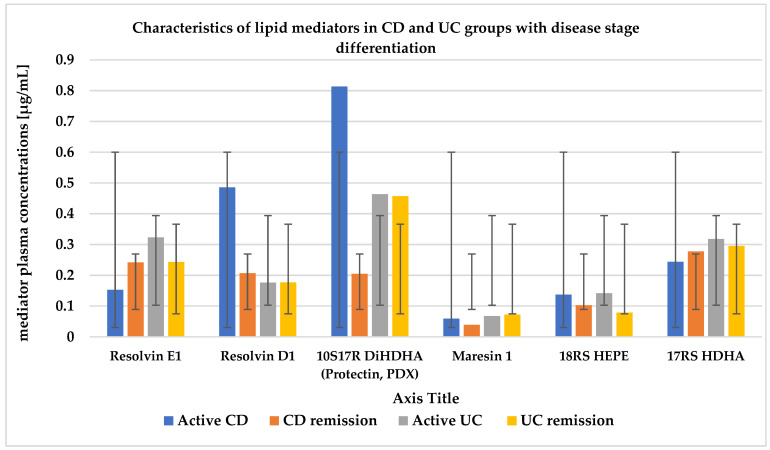
Characteristics of lipid mediators in CD and UC groups with disease stage differentiation. CD—Crohn’s disease, UC—ulcerative colitis.

When comparing the remission phase of both diseases, statistically significantly higher PDX levels (*p* = 0.014) were observed in CD relative to UC. MaR1 was also higher in UC. However, other parameters were at similar levels (Table 2 and Figure 5).

## 4. Discussion

The patterns of remissions and exacerbations of both diseases are unpredictable and the course of each disease itself is variable and dependent on the individual. Therefore, understanding the mechanism of the inflammatory remission process may provide a new therapeutic strategy for the treatment of IBD. Our study enhances the knowledge of the involvement of anti-inflammatory lipid mediators in both IBD entities. To our knowledge, there is a lack of studies on the involvement of anti-inflammatory lipid mediators derived from the EPA and DHA pathways in CD and UC. There is also no information about their involvement in the course of both disease entities. However, a diet with omega-3 acid predominance has reduced the cyclooxygenase-2 (COX-2) expression in colon and decreased the IL-6 and TNF-α production as well [16]. Additionally, the combination of omega-3 and 5-ASA has proved more beneficial in therapy, due to more effective inflammatory response inhibition by reducing NF-κB activation [17]. Moreover, as observed by other authors, most of the available animal studies are based mainly on the induction of colitis, thus referring to UC and not CD [18].

Few studies involving patients with IBD have observed changes in fatty acid metabolism in the intestinal mucosa. In a study by Pearl et al., biopsies from UC patients identified higher levels of AA and DHA and docosapentaenoic acid (DPA) and lower linoleic acid (LA) and EPA in active UC relative to UC remission, suggesting activation of synthesis pathways [19]. In another study, a significant reduction in DHA derivatives was observed in active inflammatory UC relative to UC remission. At the same time, no differences were observed with respect to EPA [9]. In contrast, in a study by Masoodi et al., EPA-derived mediators were not detected at all or dropped to an unmeasurable level in patients with active UC [20]. As shown above, the data are inconclusive. Gobbetti et al. also observed higher levels of RvD5, PD1, and 10S,17S-diHDPA in colon tissue biopsies of IBD patients relative to controls. The study by Gobbetti et al. suggests that endogenous lipid mediators such as PD1 and RvD5 from the n-3 pathway interact with intestinal inflammation by affecting the levels of pro-inflammatory cells and cytokines. Additionally, inhibition of endogenous PD1 disrupts the regulation of inflammation in the colon and leads to greater tissue damage [21]. This also points to its potential use in novel therapeutic strategies in IBD.

A study by Quiros et al. in mice showed an effect of RvE1 on cell migration and proliferation in the intestinal epithelium leading to faster healing and resolution of inflammation [22]. In a study involving adult UC patients, serum RvE1 levels were investigated. It was shown that RvE1 levels do not differ significantly in the active phase and remission of the disease, which is also what we observed in our study [23]. In contrast, analyzing the RvE1 content in the colonic mucosa of CD patients showed that RvE1 levels were significantly higher in the active phase compared to remission [24]. In another study, in mice with DSS-induced (oral solution) colitis, administration of RvE1 was shown to significantly inhibit the pro-inflammatory response of macrophages [25]. This also suggests the possibility of using RvE1 for therapeutic purposes in IBD patients. RvE1 can impair the transepithelial translocation of neutrophils and further promote the removal of neutrophils from the epithelial cell surface, which leads to the resolution of inflammation [26]. In another study on mice, RvE1 was shown to protect against the development of intestinal inflammation by inhibiting leukocyte infiltration and reducing the expression of pro-inflammatory genes [11]. In our opinion, RevE1 is most involved in the active phase in CD.

**Figure 6:**
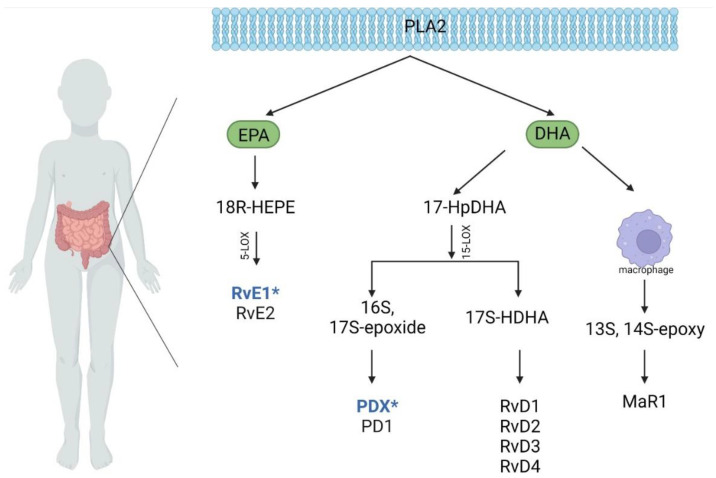
Synthesis of anti-inflammatory mediators from EPA and DHA in both disease entities (CD, UC). PLA2—phospholipase A2; EPA—eicosapentaenoic acid; DHA—docosahexaenoic acid; 18R-HEPE—18R-hydroxyeicosapentaenoic acid; 17-HpDHA—17(S)-hydroperoxy docosahexaenoic acid; 5-LOX—5-lipoxygenase; 15-LOX—15-lipoxygenase; 17S-HDHA—17-hydroxy docosahexaenoic acid; 13S, 14S—epoxy—13S, 14S-epoxy-docosahexaenoic acid; RvE—E-series resolvins; RvD—D-series resolvins; PDX—protectin DX; PD—D-protectins; Mar—maresins: Blue font—meaning in CD (active vs. remission); *—significant difference between CD and UC (active and remission); Created with BioRender.com.

In the present study, higher levels of most mediators and significantly higher levels of 10S17R DiHDHA were observed in the CD group in the active phase of the disease relative to remission. This may be related to the involvement of pro-inflammatory mediators and recruitment of anti-inflammatory mediators to quieten inflammation. Administration of 17R-HDHA and its products has been shown to have potential therapeutic effects in humans for the treatment of IBD [27]. A study on mice with colitis showed that increased availability of n-3 PUFA precursors from fish oil during feeding accelerated resolution of inflammation. However, in the resolution of inflammation itself, higher levels of the metabolite RvD1 but reduced levels of its precursor DHA were observed in colonic tissue. Additionally, lower levels of the precursor RvE and 18-HEPE were noted, which may indicate the initiation of lipid-mediated intestinal healing [28]. With reference to the results obtained by Colas et al. from the serum of healthy adults, the involvement of RvD1 in active CD can be seen, as the levels of this mediator are significantly higher. Similarly, the levels of RvE1 in our study were higher in each of the 4 groups compared to the results of the previously mentioned investigation [29]. Liu et al. observed that human neutrophils treated with PDX can inhibit COX-1 and COX-2, thereby affecting the quenching of inflammation [30]. The entire discussion of results is shown in the figure below (Figure 6).

## 5. Conclusions

RvE1 involvement in the active phase of the disease may be a marker to differentiate the two disease entities, while in the remission phase, PDX. In CD remission, PDX involvement seems to be the most important, although RvD1, MaR1 and 17RS-HEPE are also actively involved in the quenching process. In contrast, MaR1, 18RS-HEPE and RvE1 appear to be most involved in remission in UC, although the means were not significant due to large standard deviations.

Limitations of the present study may include the lack of subdivision of the group according to the drugs taken, as a result of which changes under the influence of pharmacological preparations were not taken into account. Another limitation of the study was the heterogeneity of groups with regard to disease activity. Further studies on inflammatory mediators in children and adolescents with IBD should focus on taking into account the pharmacological division and degree of activity of each disease.

## Figures and Tables

**Table 1:** Characteristics of lipid mediators between CD and UC (total).

Lipid Mediators (μg/mL)	CD *n* = 34	UC *n* = 30	*p*-Value
Resolvin E1	0.176 ± 0.11	0.306 ± 0.26	0.014 *
Resolvin D1	0.402 ± 0.76	0.174 ± 0.28	0.150
10S17R DiHDHA (Protectin, PDX)	0.625 ± 0.80	0.447 ± 0.34	0.299
Maresin 1	0.053 ± 0.06	0.067 ± 0.05	0.337
18RS HEPE	0.128 ± 0.10	0.129 ± 0.15	0.966
17RS HDHA	0.254 ± 0.21	0.306 ± 0.22	0.364

CD—Crohn’s disease; UC—ulcerative colitis; PDX—protectin DX; * statistically significant results (*p* < 0.05).

**Table 2:** Characteristics of lipid mediators in CD and UC groups with disease stage differentiation.

Lipid Mediators (μg/mL)	Active CD *n* = 24	CD Remission *n* = 10	*p*-Value	Active UC *n* = 24	UC Remission *n* = 6	*p*-Value	*p*-Value CD vs. UC Active	*p*-Value CD vs. UC Remission
Resolvin E1	0.153 ± 0.08	0.242 ± 0.16	0.167	0.323 ± 0.29	0.243 ± 0.13	0.788	0.011 *	0.958
Resolvin D1	0.486 ± 0.89	0.207 ± 0.24	0.944	0.176 ± 0.29	0.177 ± 0.23	0.864	0.137	0.713
10S17R DiHDHA (Protectin, PDX)	0.813 ± 0.91	0.205 ± 0.21	0.046 *	0.464 ± 0.35	0.457 ± 0.34	0.341	0.105	0.014 *
Maresin 1	0.059 ± 0.07	0.039 ± 0.04	0.796	0.068 ± 0.05	0.072 ± 0.05	0.864	0.585	0.156
18RS HEPE	0.137 ± 0.11	0.103 ± 0.07	0.439	0.142 ± 0.16	0.079 ± 0.04	0.213	0.906	0.564
17RS HDHA	0.244 ± 0.17	0.278 ± 0.30	0.656	0.318 ± 0.21	0.295 ± 0.27	0.643	0.217	0.713

CD—Crohn’s disease; UC—ulcerative colitis; PDX—protectin DX; * statistically significant results (*p* < 0.05).

## Data Availability

The data presented in this study are available on request from the corresponding author.
